# The Study of Microbe–Host Two-Way Communication

**DOI:** 10.3390/microorganisms10020408

**Published:** 2022-02-10

**Authors:** Famatta Perry, Ryan J. Arsenault

**Affiliations:** Department of Animal and Food Sciences, University of Delaware, Newark, DE 19716, USA; famper@udel.edu

**Keywords:** host-microbe, communication, coevolution, immunity

## Abstract

Back-and-forth intercommunication in host–pathogen interactions has long been recognized to play an important role in commensalism and microbial pathogenesis. For centuries, we have studied these microbes in our surroundings, yet many questions about the evolutionary cross-talk between host and microbe remain unanswered. With the recent surge in research interest in the commensal microbiome, basic immunological questions have returned to the fore, such as, how are vast numbers of microbes capable of coexisting within animals and humans while also maintaining a healthy functional immune system? How is the evasion and subversion of the immune system achieved by some microbes but not others? The intricate and important-to-remember two-way interaction and coevolution of host and microbe is the communication network we must tap into as researchers to answer these questions.

## 1. The Host Immune System

The vertebrate immune system is classified into two main branches, the innate and adaptive immune systems [1,2]. The innate immune system is the first line of defense against foreign invaders; therefore, innate immune cells are equipped with special “generalist” receptors that recognize molecular patterns found on microbes, known as pattern recognition receptors (PRRs) [3]. PRRs on immune cells bind to microbes, or components of microbes, and trigger immune responses. This recognition is not microbe specific and relies on the recognition motifs contained in a variety of microbes rather than a discriminating antigen. The adaptive immune system is evolutionarily more advanced and is highly discriminating between microbes. In the example of bacteria, when elimination of bacteria is not carried out by the innate PRR induced activities, both bacteria lysis and phagocytosis can be induced by opsonization facilitated by the adaptive immune system as well other adaptive responses [4,5]. Complement mediated lysis of invasive bacteria outside the cell requires the binding of specific antibodies to the target bacteria to initiate a complex capable of invading and disrupting the bacterial cell. These antibodies are produced by B-cells of the adaptive immune system. Phagocytosis can also be initiated by the binding of antibodies and acute phase proteins such as C-reactive protein and serum amyloid A and P to allow presentation on major histocompatibility complex (MHC)-II of macrophages. The binding of T-cell Receptor (TCR) to MHC-II stimulates the production of cytokines [3] thus, inducing intracellular bacteria killing mechanisms such as lysozyme activity.

There are a number of studies that describe bacterially induced changes in host immune signaling [6,7,8,9,10,11,12]. As mentioned earlier, one of the host’s first lines of defense against bacteria is via activation of PRRs. There are three primary PRRs involved in the elimination of bacteria by the innate immune system; these are Toll-like receptors (TLRs), nucleotide-binding and oligomerization (NOD)- like receptors (NLRs), and C-type lectin receptors (CLRs) [3]. Following the activation of these receptors, there is an increase in signal transduction cascades that involve mitogen-activated protein kinase (MAPK), interferon (IFN), nuclear Factor kappa-light-chain enhancer of activated B cells (NFkB)—these are other signaling proteins that induce inflammatory responses and clear the bacteria. These cascades are heavily dependent on protein kinases for activation and regulation. Later, we will discuss how these kinases can become targets for microbes to influence the host immune response.

## 2. Bacterial Response to the Host

A large proportion of immune research related to host immune–pathogen interaction focuses on the adverse outcomes during its (dys)function or how it can be manipulated when in a diseased state. This is understandable as researchers are attempting to study infections of importance with regard to disease. However, understanding the optimum functioning of the immune system in the context of a readily eliminated infectious threat (rather than a serious or chronic disease) may help us understand the basic processes of the immune system and its optimum response to microbes. For researchers studying host–pathogen interactions that result in disease states the question arises, what dynamic are we studying, an efficient immune mobilization to address an infectious agent, or a microbial beneficial response designed to evade the immune response? For example, *Salmonella* induces inflammatory responses at the epithelial layer of the gut, using the resultant permeability to invade across the gut barrier [9,13]. While the inflammatory response destroys many bacteria, it also allows invasive *Salmonella* to take hold within the host. This invasion across the endothelium is critical for the survival and spread of *Salmonella* within the host. Although it has been shown that *Salmonella* can survive in the lumen [14,15,16], it is a hostile environment for long-term survival. Some species harbor *Salmonella*, tolerate its presence and it can become part of the commensal microflora, thus facilitating its continuous shedding [17]. In other species, such as humans, the inflammatory immune responses induced by *Salmonella* triggers diarrhea which clears the contents of the lumen including nutrients, debris and bacteria [14] seems to necessitate invasion. The ability of *Salmonella* to exploit the inflammatory immune response is beneficial and critical for its survival. Infection of the epithelial or immune cells that line the lumen may result in disruption of the epithelial barrier due to the inflammation triggered by the presence of the bacteria [16], which facilitates their spread to the lamina propria. Moreover, to successfully infect organs outside of the gastrointestinal (GI) tract, *Salmonella* must cross the endothelial barrier and travel through the bloodstream. This is beneficial to the bacteria because colonizing different organs is optimal for growth and survival. This also poses a threat because crossing the endothelium into the blood causes septicemia and other fatal health complications especially in immunocompromised hosts. Who is responding to whom in this situation? Perhaps the answer to such questions eludes us because it is difficult to study the immune system when it is functioning normally and properly, or when it is in a homeostatic balance with a microbial population.

## 3. The Example of Bacteria Kinases

Host immune responses to bacteria vary partly because of the many different changes in signal transduction that can occur during exposure to pathogenic and commensal bacteria. Bacteria are capable of inducing changes not only in the host gene expression but also in the proteome by direct or indirect modification of proteins [18,19]. Here, we will focus on one example, the post-translational modification (PTM) via phosphorylation of the host proteome by bacterial kinases. Phosphorylation is the addition of a gamma phosphate from an adenosine triphosphate (ATP) molecule to a specific serine, threonine or tyrosine amino acid residue of protein [20,21,22,23,24]. This covalent process is catalyzed by enzymes known as kinases, kinases are found in both eukaryotes and prokaryotes. The word kinase is derived from the Greek word Kinein which means to move [24]. Kinases are phosphotransferases thus they move phosphate groups from one organic molecule to another, this is not limited to only proteins. Kinases can be classified into canonical kinases or pseudokinases [25]. Canonical refers to kinases that are catalytically active and pseudokinases refers to those with an evolutionary loss of function. That is, pseudokinases are inactive kinases that evolved alongside catalytically active kinases but lack key requirements to serve as kinases [26]. Research suggests that pseudokinases may play an important role in regulating other kinases [27]. Pseudokinases are also common to both eukaryotes and prokaryotes. Biologically critical kinases known to be involved in regulatory cellular processes such metabolism, immune regulation, cell maintenance, etc. are part of a large superfamily of canonical protein kinases. In eukaryotes, canonical protein kinases are divided into two main subfamilies, namely: the protein-serine/threonine kinases (STKs) and the protein-tyrosine kinases [22]. These kinases serve as on and off switches for many cellular processes. The protein-serine/threonine kinases are either membrane bound or intracellular signaling proteins that phosphorylate the oxygen of a hydroxyl (OH) group on serine or threonine amino acids. Examples of protein-serine/threonine kinases include MAPK kinases, protein kinase (A, B, C), Casein kinase, calcium/calmodulin kinases, etc. The protein-tyrosine kinases (PTKs) are either transmembrane or cytoplasmic protein kinases that phosphorylate the tyrosine residue of a protein. PTKs include receptor tyrosine kinases such as epithelial growth factor receptor (EGFR), platelet-derived growth factor receptor (PDGFR), vascular endothelial growth factor receptor (VEGFR) and non-receptor tyrosine kinases such as janus kinase (JAK), SRC, SYK family.

Kinases homologous and orthologous to the STK subfamily are found in one of the four main classes of bacterial kinases [28]. The class of bacterial kinases similar to STKs are called eukaryotic-like serine/threonine kinases (eSTKs). eSTKs are involved in a plethora of activities critical to the survival of the bacteria in a host, including the modulation of host proteome, bacterial cell wall synthesis, bacterial metabolism, and others [5] (Cozzone 2005). Studies have been shown that *Salmonella* and *Yersinia* eSTKs can directly manipulate hosts actin and cytoskeletal activity via MAP, myosin and Rho/Rac kinase activities which can alter processes such as phagocytosis, cell growth and vesicle formation in the host [9,10,29]. Kinases of *M. tuberculosis* are capable of altering metabolism activities in host cells by inhibiting the regulation of tricarboxylic acid (TCA) cycle enzymes [8,10]. Kinases in this bacteria have also been shown to decrease host inflammatory response and increase bacteria loads [7,30,31]. Another class of bacteria kinases of note is bacteria tyrosine (BY) kinase. BY kinases are kinases that phosphorylate tyrosine residues in bacteria and are not homologous to eukaryote Hanks-type tyrosine kinases, however, further studies are required to prove orthology [32,33]. The power of manipulating kinase function in health and disease has been known in human medicine for decades, a massive research and development enterprise worth many billions of dollars has focused on the inhibition of kinase activity in the treatment of cancers [34,35,36]. The fact that bacteria have taken advantage of the host use of kinase-mediated signal transduction to facilitate invasion and persistence is an excellent example of microbe–host coevolution.

**Figure 1:**
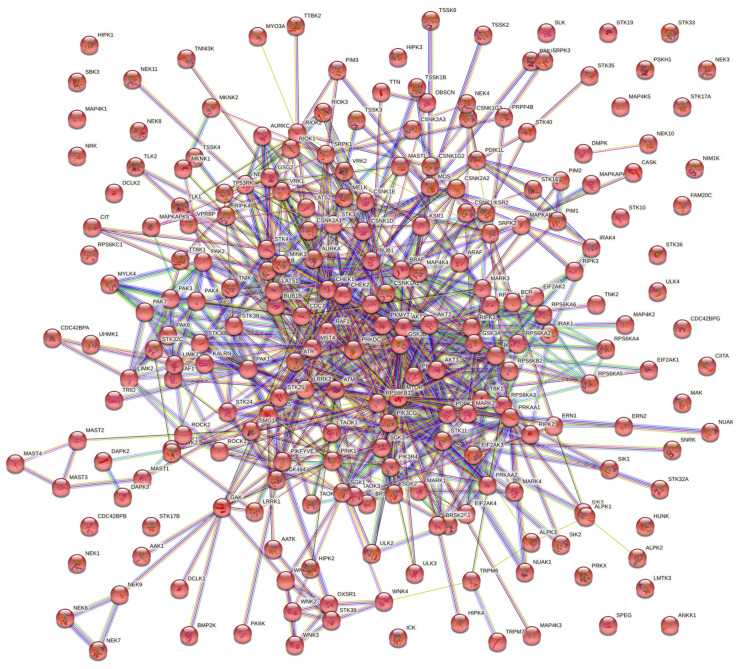
Network of protein kinases involved in immune responses in humans from STRING-database. Human protein kinases with functions in immune response were curated using UniprotKB [37]. The resulting 234 review entries were entered into STRING-database to generate the interaction network [38].

To emphasize the importance of kinases further, a uniprotKB based curation of human protein kinases that play a role in immune response resulted in 234 reviewed entries (Figure 1). Amongst these 234 kinases, there are over 200 also involved in metabolism, illustrating the indisputable importance of immunometabolism in fully understanding microbe–host interactions. The results of this curation suggests the abundance of targets of opportunity available to bacteria just in the context of kinases capable of influencing the immune system.

## 4. Microbe–Host Crosstalk and Commensals

Pathogenic bacteria interactions with the host have been studied in more detail than commensal bacteria. Thus more is understood about their kinase activity on hosts than commensal bacteria, despite commensals’ obvious benefits and intriguing effects on the immune system. Balance and homeostasis are key to a mutually beneficial coexistence between the microbiota and host. In the host, the microbiota finds a secure environment with favorable conditions such as temperature, oxygen levels and nutrient availability. The microbiota through its activities improves host metabolism, for example, by producing vitamins, and increasing nutrient availability by improving digestibility of complex carbohydrates [39,40]. A well-established microbiota competitively excludes pathogens by outcompeting pathogens for nutrients and an ecological niche within the host. The commensal microbiota provides critically important signals to the immune system for its development and maturation The abundance of microbes, mostly bacteria that reside in the intestinal tract, is evident of the complex relationship between the microbiota and the host immune system [41,42,43]. The microbiota can be harmful to the host if bacteria cross the epithelial barrier or if non-commensal microbes’ entry into the intestinal lumen is left unchecked. Changes caused by life events can disrupt the microbial niche and result in dysbiosis which may favor pathogens. Besides changes in metabolic benefits as discussed above, such disruption of the microbiome may curb the tolerogenic signals the microbiome produces, triggering proinflammatory responses that disrupt immune homeostasis. Such adverse events have been linked to diseases in many systems of hosts’ bodies other than the gut, namely, diabetes, atopic dermatitis, multiple sclerosis, asthma, etc. [44,45,46].

An example of an immune response with nuanced cross-talk between host and microbe is that of secretory immunoglobulin A (sIgA). sIgAs in the mucosal lining are important antibodies for initiating immune responses and for the competitive inhibition of bacteria binding to epithelial cells in the mucosa [47]. Study results regarding the specificity of sIgA to eliminate commensal compared to pathogenic bacteria differ. Many studies show that sIgA prevents overstimulation of the immune system to respond to bacteria that may be beneficial to the host [48]. Transportation of commensal bacteria across the Peyer’s patch M cells and dendritic cells is proposed to be essential in the host tolerance of these bacteria [49]. Tolerance is also promoted by a sIgA-commensal complex in the mucosa [48]. The exact mechanism by which the vertebrate immune system is capable of fostering such a relationship is yet to be determined. Mathias and Corthésy showed that removal of glycans found on sIgA significantly decreased its interaction with Gram-positive bacteria, indicating modification of sIgA can also alter the fate of the bacteria and the specificity of sIgA [50].

It is clear that during homeostasis, the host immune system does not respond to commensal bacteria, or certainly not in the same way as it does to pathogenic or exogenous bacteria. Perhaps, this is a result of a sort of symbiotic coevolution between the microbiota and its hosts resulting in a permanent change in physiology to facilitate tolerance of the microbiota [18]. Researchers estimate that bacteria have been coexisting with vertebrates for approximately 0.5 billion years; this dynamic interaction has shaped the microbial community and immune system, and allowed the tolerance of the microbiota by the immune system [51]. The history of innate immune cells’ development of PRR can be traced back to Cnidarians (invertebrates) need for a sort of immune specificity that distinguished symbionts from pathogens [52]. The development of the adaptive immune system corresponds to the evolution of vertebrates [53]. The oldest vertebrates need to obtain antigen recognition receptors on T-cells and B-cells as well as long lived immune memory allowed precise identification of and appropriate response to a vast majority of antigens not only as an upgrade to the host defense but more importantly, to distinguish between friends and foes, similar to their invertebrate predecessors.

## 5. Conclusions

The information provided in this paper argues that our approach to understanding the two-way communication between host and microbe in terms of health and diseases is lacking. This also highlights the difficulty associated with understanding bacteria populations where differences can exist down to the serovars of specific species [12]. The study of infectious disease is a study of both the immune response to self and the, sometimes, inappropriate or co-opted immune response. Further exploration of the immunometabolic benefits offered by coexisting with specific microbes is needed. The sequence of events that triggered the tolerance and fostering of microbial niches are yet to be understood, but the benefits of such events are undeniable. Understanding these mechanisms may allow us to treat a wide variety of infectious diseases (viral, bacterial, fungal, parasitic) as well as chronic inflammatory diseases (for example, Crohn’s, lupus, and arthritis).
